# Case Report: Staged alteplase administration under VA-ECMO support for high-risk pulmonary embolism with severe thrombocytopenia

**DOI:** 10.3389/fcvm.2026.1977983

**Published:** 2026-10-08

**Authors:** Le Kang, Yanfei Lin, Shuai Wang, Xia Ji

**Affiliations:** 1 Department of Respiratory Medicine, Linyi People's Hospital, Linyi, Shandong, China; 2 Department of Radiology, Linyi People's Hospital, Linyi, Shandong, China; 3 Emergency Department, Linyi People's Hospital, Linyi, Shandong, China; 4 Respiratory Intensive Care Unit, Linyi People's Hospital, Linyi, Shandong, China

**Keywords:** bleeding risk, hypereosinophilia, pulmonary embolism, thrombocytopenia, thrombolysis, venoarterial extracorporeal membrane oxygenation

## Abstract

High-risk pulmonary embolism (PE) with severe thrombocytopenia creates competing reperfusion and bleeding risks. A 28-year-old man presented with obstructive shock and central pulmonary arterial filling defects. The last documented platelet count before alteplase was 21 × 10⁹/L; platelet transfusion intervened, and the count at infusion initiation was unknown. Venoarterial extracorporeal membrane oxygenation (VA-ECMO) and hemostatic support were instituted. With increasing vasoactive requirements, the team selected bedside systemic thrombolysis after multidisciplinary consideration of surgical and catheter-based alternatives. Unfractionated heparin was paused at the first 20-mg alteplase infusion at 17:00. Arterial cannulation-site bleeding at 17:20 was treated with compression. The progress note documented that bleeding had stopped by 19:03; despite reduced vasoactive requirements, support dependence persisted. A second 20-mg infusion began at 19:25. Each stage lasted approximately 1 h. Heparin was restarted after the second infusion. Vasopressors were stopped on Day 1 and VA-ECMO was removed on Day 7. Hemoglobin fell from 150 to 117 g/L on Day 0 and reached 75 g/L on Day 8, prompting red-cell transfusion. Major bleeding could not be excluded. The patient was discharged on Day 19; Day 103 imaging showed no obvious residual pulmonary arterial filling defect. This case describes individualized decisions during multimodal care, not a validated alteplase regimen or evidence of its safety.

## Introduction

High-risk pulmonary embolism (PE), defined by hemodynamic instability, requires urgent evaluation for reperfusion (1, 2). Systemic thrombolysis is an option when bleeding risk is acceptable, but major bleeding and stroke remain concerns (1–3). Severe thrombocytopenia, extracorporeal membrane oxygenation (ECMO), and concurrent anticoagulation intensify this conflict (4–7). The PERT Consortium lists platelets below 100 × 10⁹/L as a relative contraindication to systemic thrombolysis (8), yet PE-specific evidence does not validate a safe threshold or regimen for severe thrombocytopenia (4, 9).

Venoarterial ECMO (VA-ECMO) supports perfusion while clinicians consider recovery or further reperfusion, but does not remove pulmonary arterial obstruction (5–7). Reduced-dose thrombolysis evidence largely concerns other PE populations (10, 11). This report describes staged alteplase administration, reassessment, and bleeding observations during VA-ECMO-supported treatment.

## Case description

### Patient information and initial presentation

A 28-year-old man (184 cm, 85 kg; body mass index 25.1 kg/m²) presented after a month of cough, five days of worsening cough and fever, and abrupt chest tightness and syncope four hours before transfer. He had received treatment for presumed pulmonary infection at another hospital. His history included allergic rhinitis and a recent pruritic rash. Day 0 denotes presentation to our hospital. At 08:21, blood pressure was 70/45 mmHg, heart rate 142 beats/min, and extremities were cold. Vasopressors and noninvasive ventilation were started (Figure 1A).

**Figure 1:**
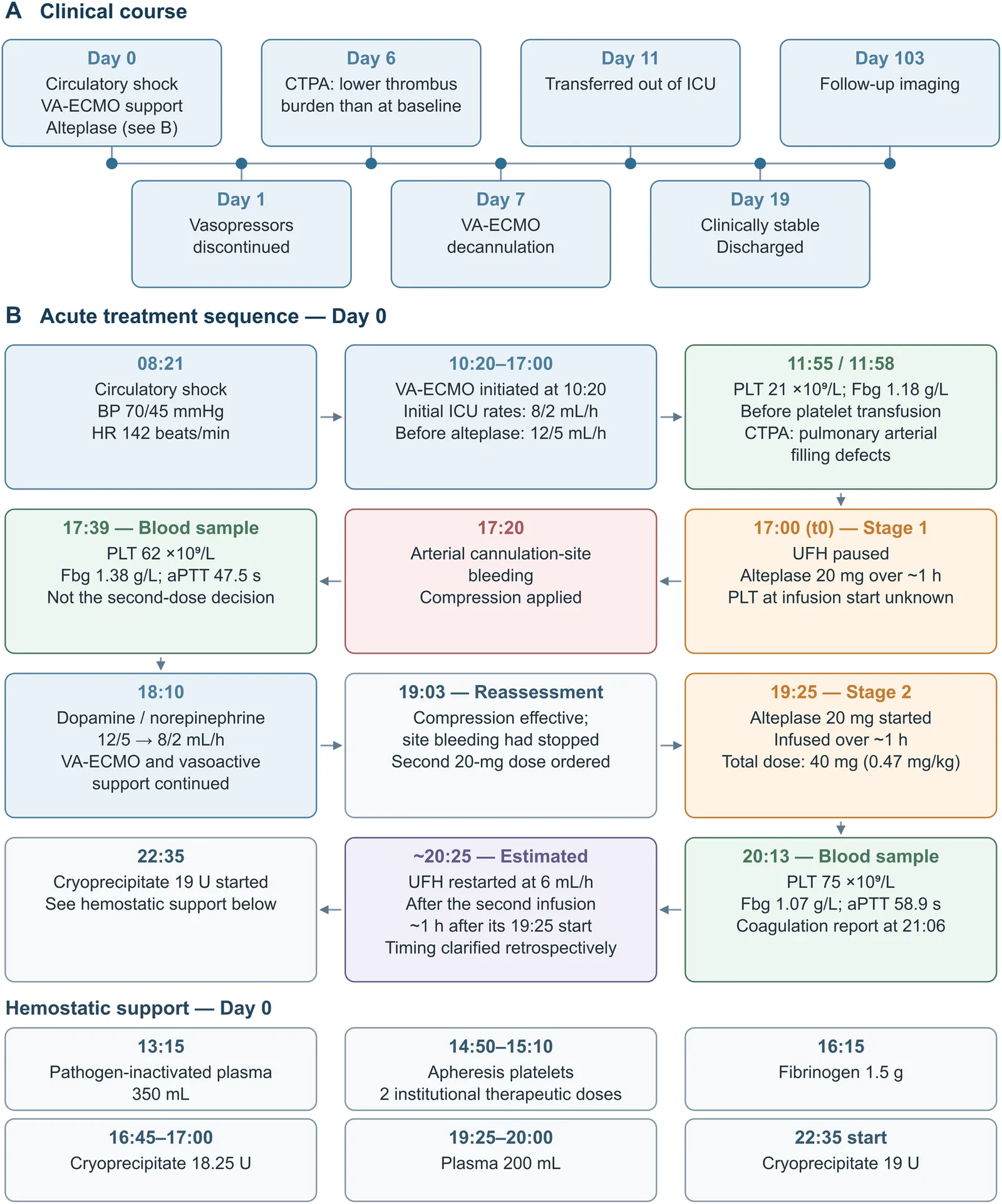
Clinical course and acute treatment sequence. **(A)** Course from Day 0 shock and VA-ECMO initiation to discharge on Day 19 and follow-up on Day 103. **(B)** VA-ECMO began at 10:20. At 17:00 (t0), UFH was paused and the first 20-mg alteplase infusion started. Arterial cannulation-site bleeding at 17:20 was treated with compression. The 17:39 values represent laboratory sampling, not the second-dose decision. Nursing records documented a reduction in dopamine/norepinephrine rates from 12/5 to 8/2 mL/h at 18:10. The progress note documented cessation of arterial cannulation-site bleeding after compression at the 19:03 reassessment. The second 20-mg dose was ordered at 19:03 and started at 19:25; UFH was resumed at 6 mL/h after its approximately 1-h infusion. Each alteplase stage lasted approximately 1 h, total 40 mg (0.47 mg/kg); exact completion timestamps and alteplase volumetric rates were unavailable. The 20:13 sample was obtained before the derived restart time, but coagulation results were not reported until 21:06. The blood-product bar summarizes nursing records. Platelets are expressed as institutional therapeutic doses and cryoprecipitate as charted units. Decision rationales and relative restart timing were clarified retrospectively. Continuous ECMO settings and definitive bleeding classification remained unavailable. Paired pump rates are reported as dopamine/norepinephrine (mL/h); preparation details, record sources, and timing limitations are provided in Table 1. BP, blood pressure; Fbg, fibrinogen; HR, heart rate; PLT, platelet count.

**Table 1:** Auditable treatment parameters and key monitoring data in this case.

Parameter	Time point	Auditable record	Interpretive boundary
Patient characteristics	Admission	28-year-old man; height, 184 cm; weight, 85 kg; body mass index, 25.1 kg/m².	Weight was used only to express the cumulative administered alteplase dose per kilogram.
Hemodynamic state	Day 0, 08:21	Blood pressure 70/45 mmHg, heart rate 142 beats/min, and cold extremities, consistent with circulatory shock.	Vasopressors and noninvasive ventilation had been started during emergency stabilization.
Acute imaging	Day 0, 09:20–11:58	At 09:20, pre-ECMO echocardiography showed right-heart enlargement, LVEF 48%, and estimated PASP 52 mmHg. Lower-limb ultrasonography showed right femoral venous thrombosis. At 11:58, post-cannulation CTPA showed multiple filling defects in the main and branch pulmonary arteries.	The sequence relative to VA-ECMO is shown explicitly; findings are reproduced from the available reports.
VA-ECMO	Day 0, 10:20	Emergency peripheral VA-ECMO through a 17-Fr left femoral arterial cannula and a 21-Fr left femoral venous cannula, with a distal limb-perfusion catheter. Unfractionated heparin 25 mg was recorded during cannulation.	Continuous ECMO settings were unavailable. Available lactate and vasoactive-rate observations are reported separately below.
Pre-alteplase laboratory values	Day 0, 11:55	WBC 22.53 × 10⁹/L, hemoglobin 150 g/L, platelets 21 × 10⁹/L, AEC 0.68 × 10⁹/L, PT 16.8 s, INR 1.38, aPTT 43.5 s, fibrinogen 1.18 g/L, D-dimer >20 mg/L FEU, and FDP >150 mg/L.	Last platelet count before transfusion; the count at the 17:00 alteplase start was unknown. This post-ECMO sample did not exclude DIC; see the diagnostic assessment in the text.
Glucocorticoids	Day 0–Day 3	Methylprednisolone 40 mg intravenously at 08:21 on Day 0, before the 08:35 blood count, then prescribed at 40 mg twice daily through Day 3. A separate dexamethasone 8-mg entry was present.	The transition between agents and cumulative glucocorticoid exposure could not be reconstructed.
Pre-alteplase support	Day 0, 11:50 to first alteplase infusion	ICU orders at 11:50, after VA-ECMO initiation at 10:20: dopamine hydrochloride 200 mg and norepinephrine bitartrate 8 mg, each added to 50 mL of 5% glucose. Nursing records showed initial ICU pump rates of 8/2 mL/h, increasing to 12/5 mL/h before alteplase. Lactate: 2.0 mmol/L at 11:55 and 3.2 mmol/L at 14:25. At 17:00, BP was 127/78 mmHg and HR 122 beats/min under support.	Drug preparations are from medication orders; pump rates are from nursing records. These are post-ECMO observations, not pre-ECMO starting doses. Final preparation volumes were not verified; rates are not converted to weight-adjusted doses.
Hemostatic support	Day 0, 13:15–17:00	Pathogen-inactivated plasma 350 mL, two institutional therapeutic doses of apheresis platelets, fibrinogen 1.5 g, and cryoprecipitate 18.25 units.	Times and quantities are taken from the nursing record; support immediately preceded and continued to t0.
First alteplase stage	Day 0, 17:00 (t0)	Unfractionated heparin was paused and the first 20-mg alteplase infusion started through right lower-limb deep venous access. The stage was infused over approximately 1 h.	Exact completion time and volumetric rate were unavailable. The 17:39 time denotes laboratory sampling, not the second-dose decision.
Interval laboratory sample	Day 0, 17:39	Platelets 62 × 10⁹/L, hemoglobin 136 g/L, fibrinogen 1.38 g/L, and aPTT 47.5 s; heart rate 122–125 beats/min and blood pressure 126–127/77–78 mmHg in the surrounding period.	Sampling time, not the second-dose decision time. The platelet rise followed transfusion and was not treated as an alteplase effect.
Vasoactive dose reduction	Day 0, 18:10	Nursing records documented dopamine/norepinephrine rates reduced from 12/5 to 8/2 mL/h.	Support requirements decreased, but VA-ECMO and vasoactive support remained necessary.
Second alteplase stage	Day 0, 19:03–19:25	At the 19:03 reassessment, the progress note documented effective compression and cessation of arterial cannulation-site bleeding. A second 20-mg order was entered at 19:03; infusion began at 19:25 and lasted approximately 1 h. Total dose across both stages: 40 mg (0.47 mg/kg).	Hemostasis is documented in the progress note. No prespecified escalation or stopping thresholds were recorded. The exact infusion-completion timestamp was unavailable.
Later laboratory sample	Day 0, 20:13	Platelets 75 × 10⁹/L, hemoglobin 117 g/L, fibrinogen 1.07 g/L, and aPTT 58.9 s; heart rate 125–126 beats/min and blood pressure 125–126/74–75 mmHg in the surrounding period.	Coagulation results were reported at 21:06 and were not known prerequisites for UFH restart. Circulation remained supported by VA-ECMO.
Further hemostatic support	Day 0 evening	Plasma 200 mL from 19:25; cryoprecipitate 19 units from 22:35. Fibrinogen was 1.53 g/L at 00:41 on Day 1.	The transfusion administration record gives cryoprecipitate completion at 22:50, whereas the transfusion progress note gives 23:00; the exact completion time remains discrepant.
Anticoagulation and transition	Day 0–Day 19	UFH was paused at first alteplase initiation. According to the treating team, it was restarted at 6 mL/h after the second infusion, approximately 1 h after its 19:25 start. The implied time of approximately 20:25 is derived, not independently recorded. The order listed 12,500 U UFH plus 50 mL saline; final preparation volume was unverified, so no U/h conversion is given. UFH was subsequently adjusted by aPTT through Day 10. Later orders included enoxaparin 4,000 anti-Xa IU every 12 h and fondaparinux 7.5 mg daily; edoxaban 60 mg daily was prescribed at discharge.	Restart interval and rate were confirmed retrospectively. The 20:13 aPTT result was reported at 21:06 and cannot be treated as a known prerequisite for restart. Patient-specific aPTT/anti-Xa targets, exact anticoagulant transition times, and reasons for each switch were not reconstructed.
Serial arterial blood gases	Day 0–Day 1	Fields: pH; PaO₂ (mmHg); PaCO₂ (mmHg); recorded FiO₂ (%); lactate (mmol/L). Day 0, 11:55: 7.369; 139; 31.7; 40; 2.0 Day 0, 14:25: 7.31; 62; 39; 41; 3.2 Day 0, 17:39: 7.35; 81; 36; 40; 2.9 Day 0, 20:13: 7.52; 211; 26; 40; 2.7 Day 1, 00:41: 7.54; 282; 34; 40; 1.7	Times denote sampling. All samples followed ECMO initiation. Recorded FiO₂ does not describe oxygenator settings; these measurements cannot isolate the effect of pulmonary reperfusion.
Platelet follow-up	Day 0–Day 10	Platelet counts (×10⁹/L; sampling times): Day 0: 17:39, 62; 20:13, 75. Days 1–3: 05:19, 65; 05:30, 64; 05:08, 77, respectively. Day 4: 05:09, 91; 18:38, 54. Day 5: 06:13, 74; 18:27, 81. Days 6–10: 05:25, 87; 05:31, 119; 05:53, 129; 05:07, 173; 06:01, 160, respectively.	Two institutional therapeutic doses of apheresis platelets were transfused on Day 0, 14:50–15:10. No additional platelet transfusions were documented in the available Day 1–10 transfusion records and temporary medication orders. Day 8 transfusion was 2 U of red cells, not platelets. Counts fluctuated before recovery; this does not definitively exclude HIT.
Concurrent prothrombotic findings	Day −3–Day 103	AEC 3.16 × 10⁹/L (Day −3), 2.93 × 10⁹/L (Day 0, 08:35), 0.68 × 10⁹/L (11:55), 1.53 × 10⁹/L (Day 43), and 0.47 × 10⁹/L (Day 103); homocysteine 54 μmol/L on Day 1.	The numerical criterion for hypereosinophilia was met, but organ attribution, alternative prothrombotic factors, and etiologic assessment were insufficient for a diagnosis of hypereosinophilic syndrome.
Echocardiographic and CTPA course	Day 1–Day 103	Estimated PASP decreased from 45 mmHg on Day 1 to 28 mmHg on Day 16; TAPSE was 18–19 mm. CTPA on Day 6 showed a lower thrombus burden than at baseline, and CTPA on Day 103 showed no obvious residual filling defect.	Changes occurred after VA-ECMO, anticoagulation, alteplase infusions, and multimodal supportive treatment and are not assigned to a single intervention.
Safety observations	Acute hospitalization	Arterial cannulation-site bleeding at 17:20 on Day 0 was treated with compression; cessation was documented in the 19:03 progress note. Hb fell from 150 to 136 and 117 g/L on Day 0 and reached 75 g/L on Day 8. Two units of leukocyte-reduced red cells were transfused on Day 8, 10:45–12:45, for a continuing Hb decline in a critically ill patient. No intracranial or gastrointestinal hemorrhage was documented.	The 33-g/L Day 0 hemoglobin decline met numerical major-bleeding thresholds, but bleeding attribution and the relation to Day 8 transfusion remained uncertain; the event was not classified as confirmed non-major bleeding.
Clinical outcome	Day 1–Day 103	Vasopressors were stopped on Day 1, VA-ECMO was removed on Day 7, the patient left intensive care on Day 11, and he survived to discharge on Day 19. Day 103 CTPA, echocardiography, and lower-limb ultrasonography were reported without obvious abnormality.	Actual vasopressor cessation on Day 1 and later order reconciliation on Day 3 were clarified by the treating team. Day 103 echocardiography did not report PASP. Ventilation–perfusion imaging and right-heart catheterization were not performed; CTEPH was not formally excluded.

Day 0 denotes presentation to our hospital; t0 is first alteplase initiation at 17:00. Times are medication-event or sampling times unless stated otherwise. Vasoactive preparations are from medication orders, pump-rate changes from nursing records, and 19:03 hemostasis from the progress note. UFH restart timing was clarified retrospectively; approximately 20:25 is derived, not an independently recorded timestamp. Platelet count at first alteplase initiation was unknown after intervening transfusion. Apheresis platelets are reported as institutional therapeutic doses and cryoprecipitate as charted units. Each alteplase stage lasted approximately 1 h; exact completion timestamps and volumetric rates were unavailable. The treatment sequence was individualized, not a validated dosing algorithm.

AEC, absolute eosinophil count; aPTT, activated partial thromboplastin time; BP, blood pressure; CTEPH, chronic thromboembolic pulmonary hypertension; CTPA, computed tomography pulmonary angiography; DIC, disseminated intravascular coagulation; FDP, fibrin degradation products; FEU, fibrinogen-equivalent units; FiO₂, fraction of inspired oxygen; Hb, hemoglobin; HR, heart rate; ICU, intensive care unit; INR, international normalized ratio; LVEF, left ventricular ejection fraction; PaCO₂, arterial carbon dioxide partial pressure; PaO₂, arterial oxygen partial pressure; PASP, pulmonary artery systolic pressure; PLT, platelet count; PT, prothrombin time; TAPSE, tricuspid annular plane systolic excursion; HIT, heparin-induced thrombocytopenia; UFH, unfractionated heparin; VA-ECMO, venoarterial extracorporeal membrane oxygenation; WBC, white blood cell count.

Platelets fell from 85 × 10⁹/L on Day −3 to 21 × 10⁹/L at 08:35 on Day 0, before ECMO. Further verification established that heparin had not been used during the recent outside-hospital admission. The platelet decline preceded the first heparin administration at our institution, a temporal sequence that does not support prior-heparin-associated heparin-induced thrombocytopenia (HIT) as the explanation for the initial thrombocytopenia. No formal 4Ts score was documented, and platelet factor 4 (PF4)/heparin antibody and functional testing were not performed; HIT was therefore not definitively excluded.

### Diagnostic assessment

At 09:20, echocardiography showed right atrial and ventricular enlargement, left ventricular ejection fraction (LVEF) 48%, estimated pulmonary artery systolic pressure (PASP) 52 mmHg, moderate tricuspid regurgitation, and a 14 × 9-mm echogenic focus at the pulmonary artery bifurcation. Ultrasonography identified right femoral venous thrombosis. After bedside ECMO-team assessment, VA-ECMO was initiated at 10:20 for progressive circulatory failure. Computed tomography pulmonary angiography (CTPA) after initial stabilization at 11:58 showed filling defects in the main and branch pulmonary arteries (Figures 2A,D). The echogenic focus was not specifically reassessed later.

**Figure 2:**
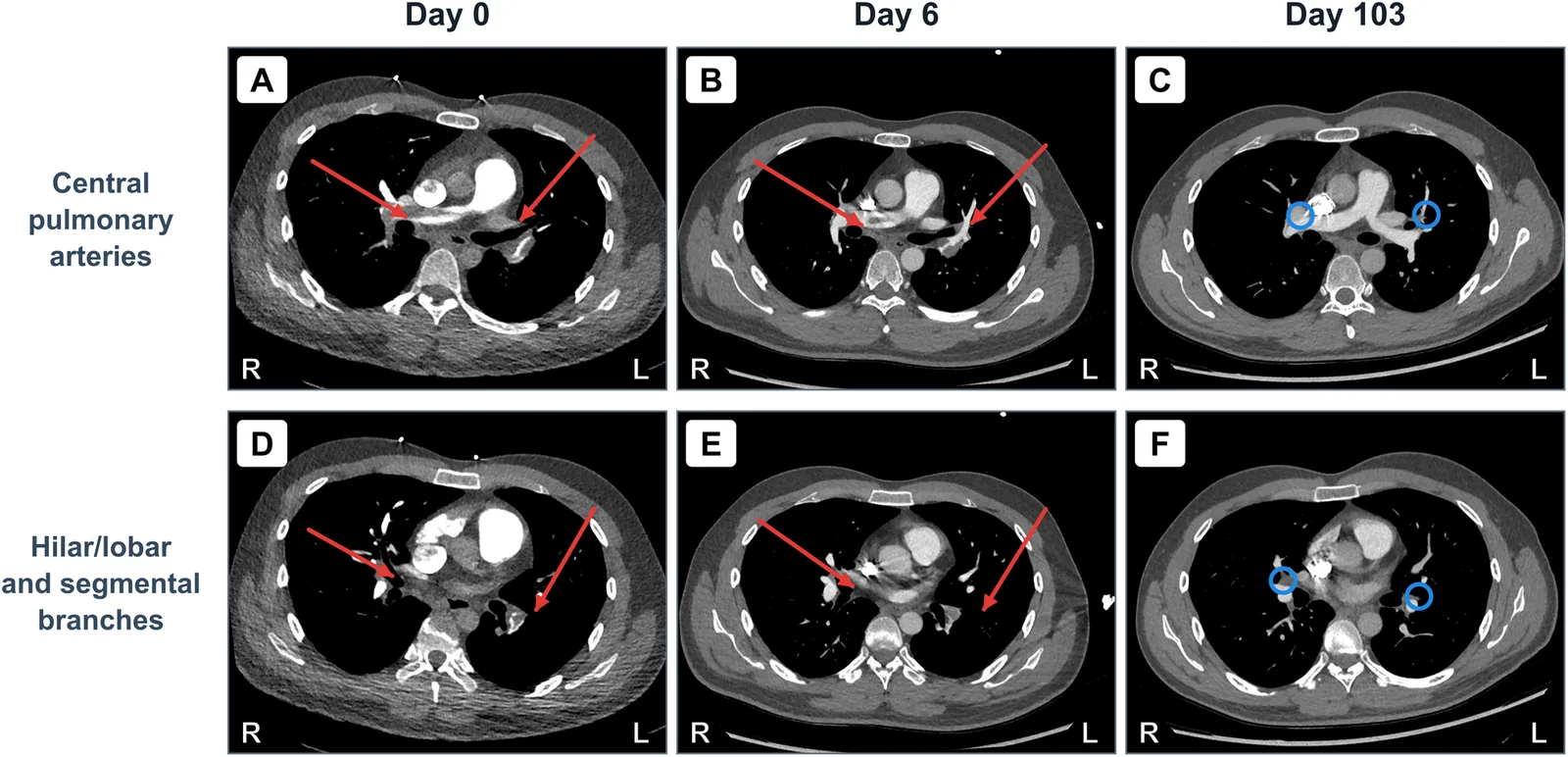
Serial computed tomography pulmonary angiography at three time points. **(A–C)** Representative central pulmonary arterial images on Day 0, Day 6, and Day 103, respectively. **(D–F)** Representative images at the hilar/lobar and segmental branch levels at the same time points. Baseline CTPA **(A,D)** showed multiple filling defects in the central and bilateral branch pulmonary arteries. After multimodal treatment, Day 6 images **(B,E)** showed a lower thrombus burden than at baseline. On Day 103 **(C,F)**, no evident filling defects were seen at the corresponding pulmonary arterial sites. Red solid arrows indicate filling defects; blue open circles identify the corresponding vascular sites at follow-up. Representative pulmonary arterial levels were selected for each examination. CTPA, computed tomography pulmonary angiography.

At 11:55, platelets were 21 × 10⁹/L, hemoglobin 150 g/L, prothrombin time (PT) 16.8 s, international normalized ratio (INR) 1.38, activated partial thromboplastin time (aPTT) 43.5 s, and fibrinogen 1.18 g/L. D-dimer exceeded 20 mg/L fibrinogen-equivalent units (FEU) and fibrin degradation products (FDP) exceeded 150 mg/L. Complete laboratory values and serial arterial blood gases are provided in Table 1.

Hematology reviewed the progressive thrombocytopenia on Day 0 and documented an uncertain cause. Acute thrombosis/critical-illness-related consumption, immune or infectious causes, and drug-related thrombocytopenia were considered in the differential. Available autoimmune screening was unrevealing, but did not exclude all alternative causes. Although the treating team retrospectively reported that disseminated intravascular coagulation (DIC) had not been favored, no formal score was documented. The 11:55 findings could meet the traditional International Society on Thrombosis and Haemostasis (ISTH) overt-DIC laboratory threshold if the D-dimer elevation were scored as strong: platelets contributed 2 points and fibrin-related markers would contribute 3. Fibrinogen of 1.18 g/L contributed 0 points; the PT component could not be assigned without the laboratory control value. DIC therefore could not be excluded. This sample was obtained after ECMO initiation, limiting interpretation of the pretreatment coagulation state but not invalidating the laboratory findings. The cause of thrombocytopenia remained unresolved, and ECMO cannot explain the decline that preceded cannulation.

Antinuclear, anti-double-stranded DNA, antineutrophil cytoplasmic, anti-glomerular basement membrane, anticardiolipin, and anti-*β*₂-glycoprotein I antibody testing was unrevealing. The normalized lupus anticoagulant ratio was 1.12. Protein C and antithrombin activity were within reference ranges; protein S was 74% (reference 75%–130%), with interpretation limited by acute thrombosis and anticoagulation. BCR::ABL1 testing was negative; bone marrow and tissue examinations were not performed. Absolute eosinophil count (AEC) was 3.16 × 10⁹/L on Day −3, 2.93 × 10⁹/L at 08:35 on Day 0, and 0.68 × 10⁹/L at 11:55 after glucocorticoids. Homocysteine was 54 μmol/L on Day 1.

### Therapeutic intervention

Peripheral VA-ECMO used a 17-Fr femoral arterial and a 21-Fr femoral venous cannula, with distal perfusion. Cannulation unfractionated heparin (UFH) was charted as 25 mg; corresponding activity units were unavailable. The team selected UFH for anticoagulation during acute PE treatment and VA-ECMO support because it could be titrated to coagulation measurements and bleeding, had a short duration of action, and could be stopped and reversed with protamine if necessary. Methylprednisolone 40 mg was given at 08:21 and prescribed twice daily through Day 3. A separate dexamethasone entry prevented complete reconstruction of glucocorticoid exposure.

Vasoactive therapy had begun during emergency stabilization at 08:21. The ICU medication orders entered at 11:50, after VA-ECMO initiation at 10:20, listed dopamine hydrochloride 200 mg and norepinephrine bitartrate 8 mg, each added to 50 mL of 5% glucose. Nursing records documented dopamine/norepinephrine rates of 8/2 mL/h during this initial ICU treatment period, increasing to 12/5 mL/h before the first alteplase infusion. Final preparation volumes were not independently verified, so the recorded pump rates and preparation orders are reported without conversion to weight-adjusted doses. Post-ECMO lactate rose from 2.0 mmol/L at 11:55 to 3.2 mmol/L at 14:25. These findings raised concern about persistent support dependence.

The treating team retrospectively confirmed multidisciplinary consideration of surgical embolectomy, catheter-based thrombectomy, and systemic thrombolysis. Surgical trauma and perioperative bleeding were major concerns in the setting of severe thrombocytopenia. Catheter-based treatment required transfer to an operating room. The team recalled marked intolerance of even minor movement, with a sense of impending death, both before and after VA-ECMO initiation. Although CTPA had been completed after initial stabilization, concern about further circulatory deterioration during subsequent transfer remained. Bedside systemic thrombolysis was therefore selected after an individualized risk–benefit assessment.

The initial 20-mg alteplase dose was selected by individual clinical judgment because of the competing risks of persistent pulmonary arterial obstruction and bleeding. The intention was to assess the clinical response and bleeding before deciding whether to administer a further dose. This was not a guideline-defined regimen or a documented institutional protocol; expressing the total dose as 0.47 mg/kg describes exposure rather than the basis for dose selection.

Hematology recommended platelet transfusion according to bleeding and serial blood counts; the transfusion note documented cryoprecipitate for coagulation-factor replacement. Two institutional therapeutic doses of apheresis platelets were transfused from 14:50 to 15:10, with plasma, fibrinogen, and cryoprecipitate support before alteplase. No target platelet or fibrinogen threshold was documented, and no platelet count was obtained after transfusion but before the first infusion. The rise from 21 to 62 × 10⁹/L was interpreted primarily in relation to platelet transfusion, not as spontaneous recovery or an alteplase effect.

At 17:00 (t0), UFH was paused and the first 20-mg alteplase infusion began through right lower-limb deep venous access (Figures 1B). Arterial cannulation-site bleeding occurred at 17:20 and was managed with compression.

The 17:39 sample showed platelets of 62 × 10⁹/L, fibrinogen of 1.38 g/L, and aPTT of 47.5 s after hemostatic support. Nursing records documented a reduction in dopamine and norepinephrine rates to 8 and 2 mL/h at 18:10. The progress note documented effective compression and cessation of arterial cannulation-site bleeding at the 19:03 reassessment. Despite initial improvement, VA-ECMO and vasoactive support remained necessary. The team judged that further reperfusion might provide benefit after bleeding control and ordered another 20 mg at 19:03; infusion began at 19:25. No prespecified numerical escalation or stopping criteria were documented. Each stage lasted approximately 1 h; the total administered dose was 40 mg (0.47 mg/kg). Exact completion timestamps and volumetric alteplase rates were unavailable.

UFH was restarted at 6 mL/h after the second alteplase infusion, approximately 1 h after its 19:25 start, according to the treating team. The implied time of approximately 20:25 is not an independent execution timestamp. Preparation details and limitations are reported in Table 1. UFH was subsequently adjusted by aPTT through Day 10. Later orders included enoxaparin 4,000 anti-Xa IU every 12 h and fondaparinux 7.5 mg daily; discharge anticoagulation was edoxaban 60 mg daily. The patient-specific aPTT or anti-Xa target, exact transition times, and reasons for each switch could not be reconstructed. Additional plasma and cryoprecipitate were given; fibrinogen was 1.53 g/L at 00:41 on Day 1.

### Follow-up and outcomes

The treating team confirmed that vasopressors were discontinued on Day 1; formal order discontinuation on Day 3 reflected subsequent order reconciliation. Repeat CTPA on Day 6 showed a lower pulmonary arterial thrombus burden than at baseline, although residual filling defects remained (Figures 2B,E). VA-ECMO was removed on Day 7 after a brief reduction and clamp assessment; the operative record documented 50 mL of blood loss.

The treating team reported no pleural or abdominal fluid collection or visible retroperitoneal hematoma on bedside ultrasonography during the bleeding assessment. The patient remained alert without neurological signs suggestive of intracranial hemorrhage; these observations did not exclude occult bleeding. Hemoglobin decreased from 150 g/L at 11:55 to 136 g/L at 17:39 and 117 g/L at 20:13 on Day 0. On Day 8, hemoglobin was 75 g/L, and two units of leukocyte-reduced red cells were transfused from 10:45 to 12:45 for a continuing hemoglobin decline in a critically ill patient.

Platelet counts fluctuated during continued UFH treatment: 65 and 64 × 10⁹/L on Days 1 and 2, 91 then 54 × 10⁹/L on Day 4, and 129, 173, and 160 × 10⁹/L on Days 8–10, respectively (Table 1). No additional platelet transfusions were documented in the available transfusion records and temporary medication orders from Day 1 through Day 10. This trajectory indicates eventual recovery, not a continuous rise or definitive exclusion of HIT.

Estimated PASP was 45 mmHg on Day 1 and 28 mmHg on Day 16. The patient left intensive care on Day 11 and was discharged on Day 19 on edoxaban 60 mg and folic acid 1.6 mg daily; no glucocorticoid was listed. AEC was 1.53 × 10⁹/L on Day 43 and 0.47 × 10⁹/L on Day 103. Day 103 CTPA showed no obvious residual filling defect; echocardiography and lower-limb ultrasonography were reported without obvious abnormality (Figures 2C,F). Ventilation–perfusion scanning and right-heart catheterization were not performed.

## Discussion

### VA-ECMO-supported reperfusion under competing risks

This case describes individualized reperfusion decisions during VA-ECMO support in a patient with severe thrombocytopenia. The platelet count of 21 × 10⁹/L preceded transfusion and must not be treated as the count at alteplase initiation.

VA-ECMO can stabilize perfusion and unload the failing right ventricle, but it does not directly remove pulmonary arterial obstruction. Evidence for adding reperfusion therapy during VA-ECMO remains observational and inconsistent. In a Japanese nationwide cohort of 1,220 patients, systemic thrombolysis within two days of VA-ECMO was not associated with lower in-hospital mortality; a sensitivity analysis restricted to monteplase showed more major bleeding (12). A three-center cohort likewise found no difference in 90-day survival or severe hemorrhage between patients cannulated after failed thrombolysis and those who had not received thrombolysis, although the comparison was strongly affected by treatment selection (13). The ELSO registry describes substantial mortality across ECMO modes but cannot determine the effect of a particular reperfusion strategy (14). More recently, early mechanical reperfusion during VA-ECMO was not associated with a statistically significant reduction in 90-day mortality in a 39-center cohort, and bleeding occurred in half of the patients (15). Taken together, these cohorts define the uncertainty within which individual decisions are made rather than a preferred treatment combination.

Baran et al. described a single-center series of 27 high-risk PE patients in which ECMO was considered alongside surgical or catheter-based reperfusion, with initiation tailored to the perioperative course (16). This experience illustrates ECMO as support for a treatment pathway rather than a substitute for selecting a reperfusion strategy. Mechanical clot removal, continued ECMO with anticoagulation, and bedside systemic thrombolysis entail different procedural, transport, and bleeding risks.

A randomized trial compared 50 mg with 100 mg over 2 h, but did not study severe thrombocytopenia during VA-ECMO (17). A retrospective comparison found more rescue treatment after 50 mg without a clear bleeding advantage (18). Other studies used heterogeneous regimens and populations (10, 11). Differences in populations and regimens mean that these studies provide only indirect context for the staged 20-mg doses used in this case.

A 2022 systematic review identified 11 cases of PE with thrombocytopenia, with inconsistent platelet sampling times and no comparative evidence (4). Later reviews reached similar conclusions (9). Selected reports involved malignancy, thrombotic thrombocytopenic purpura, HIT, lymphoma, and postpartum antiphospholipid syndrome (19–24). ECMO was not reported in the direct cases summarized in Table 2. These selected experiences cannot define a safe platelet threshold and are susceptible to publication and survivor bias (4, 9).

**Table 2:** Selected directly relevant reports of systemic thrombolysis for pulmonary embolism with thrombocytopenia.

Report	Design	Clinical context and high-risk presentation	Pre-thrombolysis or nearest platelet count (×10⁹/L)	Systemic thrombolysis	ECMO	Bleeding and outcome
Ata et al., 2022 (4)	Systematic review	Eleven published cases of PE with thrombocytopenia.	Heterogeneous timepoints; not pooled here	Alteplase in 10 cases; urokinase in 1.	Not summarized at case level	No thrombocytopenia-related complication reported; one death from aspiration pneumonia.
Burns and McLaren, 2009 (19)	Case report and systematic review	77-year-old man; HIT, catheter-related right atrial thrombus, and hemodynamically significant PE.	73	rt-PA 100 mg over 2 h.	Not reported	No bleeding complication reported; right atrial thrombus resolved and the patient survived.
Alirezaei et al., 2018 (20)	Case report	65-year-old woman; metastatic breast cancer with brain metastasis and shock.	50 (60 at admission)	Alteplase 100 mg over 2 h.	Not reported	No bleeding reported; platelets were transfused after a later fall to 23 × 10⁹/L; discharged alive.
Zhu et al., 2015 (21)	Case report	33-year-old woman; thrombotic thrombocytopenic purpura and PE-related cardiac arrest.	23	rt-PA 5-mg intravenous bolus followed by a 45-mg infusion.	Not reported	No bleeding reported; return of spontaneous circulation and survival.
Hourmouzis et al., 2015 (22)	Case report	56-year-old woman; postoperative HIT and high-risk PE.	63	Systemic rt-PA 100 mg.	Not reported	No major bleeding reported; hemodynamic improvement and survival.
Iqbal et al., 2023 (23)	Case report	42-year-old woman; lymphoma, hypotension, and recurrent cardiac arrest.	6	Alteplase 50 mg intravenously over approximately 10 min.	Not reported	Mild-to-moderate nasal and oral bleeding with platelet transfusion; weaned from ventilation and survived.
Ahmadzadeh et al., 2025 (24)	Case report	34-year-old postpartum woman; antiphospholipid syndrome and blood pressure 86/60 mmHg.	48 (result returned near the end of thrombolysis)	Alteplase 100 mg over 2 h.	Not reported	No in-hospital bleeding; discharged on Day 9.
Boca et al., 2026 (9)	Case report and qualitative review	34-year-old postpartum woman; suspected HIT, hypotension, and norepinephrine requirement.	53	Alteplase 10-mg bolus followed by 40-mg and 50-mg infusions; total 100 mg.	Not reported	No major bleeding; right-heart and hemodynamic improvement; discharged alive.
Present case	Case report	28-year-old man; high-risk PE with obstructive shock and severe thrombocytopenia before platelet transfusion.	21 at 11:55 on Day 0, before platelet transfusion; count at alteplase initiation unknown.	Two 20-mg alteplase stages, started at 17:00 and 19:25 with interim reassessment; each was infused over approximately 1 h, for a cumulative administered dose of 40 mg (0.47 mg/kg).	VA-ECMO	Arterial cannulation-site bleeding at 17:20; the progress note documented cessation after compression at 19:03. Substantial Hb decline and Day 8 red-cell transfusion. Major bleeding could not be excluded; discharged on Day 19.

Selected directly relevant adult reports were identified through a focused PubMed search and reference chaining updated through 21 August 2026. This narrative comparison places the present case in context and is not an exhaustive systematic review. Reports were prioritized when pulmonary embolism was objectively diagnosed, thrombocytopenia was present, and systemic thrombolysis was administered; pediatric cases, catheter-directed thrombolysis, and rt-PA used for indications other than pulmonary embolism were not considered. The reviews by Ata et al., Burns and McLaren, and Boca et al. had different scopes; review totals were not added to the individually listed cases. ECMO is shown as not reported when the source did not describe its use. Platelet counts were extracted at the nearest documented pre-thrombolysis timepoint where possible. In the present case, two institutional therapeutic doses of apheresis platelets were transfused between the last documented count and alteplase initiation; 21 × 10⁹/L must not be interpreted as the post-transfusion count at treatment initiation.

ECMO, extracorporeal membrane oxygenation; Hb, hemoglobin; HIT, heparin-induced thrombocytopenia; PE, pulmonary embolism; rt-PA, recombinant tissue-type plasminogen activator; VA-ECMO, venoarterial extracorporeal membrane oxygenation.

### Treatment sequence and bleeding risk

The post-transfusion platelet rise does not establish spontaneous recovery or an alteplase effect. Neither platelet nor fibrinogen values were prespecified dose-escalation triggers. Support requirements decreased after the first stage, and the decision to administer the second stage depended on clinical reassessment rather than demonstrated failure of the first dose.

The Day 0 hemoglobin decline of 33 g/L exceeded the numerical ISTH major-bleeding threshold and fell within the BARC 3a hemoglobin range. However, the proportion attributable to bleeding and the relationship between the cannulation-site event and Day 8 transfusion could not be established; dilution, hemolysis, and phlebotomy were not quantified. We therefore report a potentially major bleeding event without assigning a definitive retrospective grade, rather than classifying it as confirmed non-major bleeding (25, 26).

### Hypereosinophilia and alternative prothrombotic factors

The eosinophil counts of 3.16 and 1.53 × 10⁹/L, 46 days apart, met the numerical and confirmatory interval requirements for hypereosinophilia (27). Hypereosinophilic syndrome (HES) was not established because eosinophil-attributable organ injury and an adequate etiologic evaluation were lacking. BCR::ABL1 testing was negative, but this does not exclude all clonal eosinophilic disorders.

Homocysteine was 54 μmol/L on a single acute-phase measurement. The HOPE-2 VTE analysis found no reduction in VTE with homocysteine-lowering vitamin therapy (28), but did not resolve causality at this patient's concentration. Without repeat measurements and folate/vitamin B12 assessment, neither finding establishes the cause of PE.

### Follow-up, limitations, and clinical implications

Day 103 imaging was reassuring but did not formally exclude chronic thromboembolic pulmonary hypertension because ventilation–perfusion imaging, right-heart catheterization, and structured symptom assessment were unavailable (1, 2).

Continuous ECMO blood flow, pump speed, sweep-gas flow, and oxygenator gas oxygen fraction records were unavailable, and serial quantitative right ventricular measurements around thrombolysis could not be reconstructed. Available lactate and blood gas values were obtained under extracorporeal and respiratory support. These gaps prevent reconstruction of changes in support intensity and limit attribution of physiological improvement to reperfusion.

This retrospective single-case report cannot separate the effects of alteplase from VA-ECMO, anticoagulation, and hemostatic support. Treatment rationales clarified retrospectively by the team are distinguished from contemporaneous observations. Missing continuous ECMO settings, incomplete anticoagulation details, and uncertain bleeding attribution limit interpretation. The case illustrates individualized reassessment under competing risks; it neither establishes the efficacy or safety of staged alteplase nor supports 20 mg followed by 20 mg as a general dosing regimen.

## Patient perspective

At follow-up, the patient reported limited recollection of the initial resuscitation and VA-ECMO treatment. After learning how critical his condition had been, he expressed relief about his recovery. He described a gradual return to his usual daily activities after discharge and was satisfied with his progress at the three-month follow-up. He agreed to share his experience in the hope that it might help patients and clinicians facing similarly difficult treatment decisions.

## Data Availability

The de-identified clinical data supporting this case report are included in the article. The underlying medical records and complete imaging data are not publicly available or available on request because of patient confidentiality and the risk of re-identification. Any exceptional access would require institutional and ethics approval and compliance with the patient's consent. Requests to access the datasets should be directed to shuaiwang619@gmail.com.

## References

[1] Konstantinides SV Meyer G Becattini C Bueno H Geersing G-J Harjola V-P. 2019 ESC guidelines for the diagnosis and management of acute pulmonary embolism developed in collaboration with the European Respiratory Society (ERS). Eur Heart J. (2020) 41:543–603. 10.1093/eurheartj/ehz405 31504429

[2] Creager MA Barnes GD Giri J Mukherjee D Jones WS Burnett AE. 2026 AHA/ACC/ACCP/ACEP/CHEST/SCAI/SHM/SIR/SVM/SVN guideline for the evaluation and management of acute pulmonary embolism in adults: a report of the American College of Cardiology/American Heart Association Joint Committee on Clinical Practice Guidelines. Circulation. (2026) 153:e977–e1051. 10.1161/CIR.0000000000001415 41712677

[3] Meyer G Vicaut E Danays T Agnelli G Becattini C Beyer-Westendorf J. Fibrinolysis for patients with intermediate-risk pulmonary embolism. N Engl J Med. (2014) 370:1402–11. 10.1056/NEJMoa1302097 24716681

[4] Ata F Hamad Ibrahim W Nasser Affas M Ahmad Khan H Younas HW Maat Z. Safety and outcomes of thrombolytic therapy in patients with pulmonary embolism and thrombocytopenia: a systematic review. Qatar Med J. (2022) 2022:33. 10.5339/qmj.2022.33 35974889 PMC9372479

[5] Meneveau N Guillon B Planquette B Piton G Kimmoun A Gaide-Chevronnay L. Outcomes after extracorporeal membrane oxygenation for the treatment of high-risk pulmonary embolism: a multicentre series of 52 cases. Eur Heart J. (2018) 39:4196–204. 10.1093/eurheartj/ehy464 30137303

[6] Boey JJE Dhundi U Ling RR Chiew JK Fong NC-J Chen Y. Extracorporeal membrane oxygenation for pulmonary embolism: a systematic review and meta-analysis. J Clin Med. (2024) 13:64. 10.3390/jcm13010064 PMC10779708 38202071

[7] Cardona S Downing JV Witting MD Haase DJ Powell EK Dahi S. Venoarterial extracorporeal membrane oxygenation with or without advanced intervention for massive pulmonary embolism. Perfusion. (2024) 39:665–74. 10.1177/02676591231177909 37246150

[8] Rivera-Lebron B McDaniel M Ahrar K Alrifai A Dudzinski DM Fanola C. Diagnosis, treatment and follow up of acute pulmonary embolism: consensus practice from the PERT consortium. Clin Appl Thromb Hemost. (2019) 25:1076029619853037. 10.1177/1076029619853037 31185730 PMC6714903

[9] Boca MI Cozlac A-R Streian CG Crisan S Lazar M-A Virtosu M-D. Thrombolytic therapy in high-risk pulmonary embolism with thrombocytopenia: case report and literature review. J Clin Med. (2026) 15:2569. 10.3390/jcm15072569 41976869 PMC13073970

[10] Sharifi M Bay C Skrocki L Rahimi F Mehdipour M; MOPETT Investigators. Moderate pulmonary embolism treated with thrombolysis from the MOPETT trial. Am J Cardiol. (2013) 111:273–7. 10.1016/j.amjcard.2012.09.027 23102885

[11] Rojas Murguia A Mukherjee D Ojha C Rajachandran M Siddiqui TS Nickel NP. Reduced-dose thrombolysis in acute pulmonary embolism: a systematic review. Angiology. (2024) 75:208–18. 10.1177/00033197231167062 37060258

[12] Nishimoto Y Ohbe H Matsui H Nakajima M Sasabuchi Y Sato Y. Effectiveness of systemic thrombolysis on clinical outcomes in high-risk pulmonary embolism patients with venoarterial extracorporeal membrane oxygenation: a nationwide inpatient database study. J Intensive Care. (2023) 11:4. 10.1186/s40560-023-00651-w 36740697 PMC9901114

[13] Levy D Saura O Passarelli MT Lucenteforte M Lebreton G Bougle A. Thrombolysis before venoarterial ECMO for high-risk pulmonary embolism: a retrospective cohort study. Intensive Care Med. (2024) 50:1287–97. 10.1007/s00134-024-07501-9 38913095

[14] Rivers J Pilcher D Kim J Bartos JA Burrell A. Extracorporeal membrane oxygenation for the treatment of massive pulmonary embolism: an analysis of the ELSO database. Resuscitation. (2023) 191:109940. 10.1016/j.resuscitation.2023.109940 37625576

[15] Levy D Petit M Assouline B Dietl A Lebreton G Giraud R. Early mechanical reperfusion in high-risk pulmonary embolism supported by venoarterial extracorporeal membrane oxygenation: a multicenter international cohort study. Am J Respir Crit Care Med. (2026) 212:474–86. 10.1093/ajrccm/aamaf116 41738098

[16] Baran C Kayan A Baran CS. Algorithm of high-risk massive pulmonary thromboembolism with extracorporeal membrane oxygenation. J Clin Med. (2024) 13:6822. 10.3390/jcm13226822 39597964 PMC11594580

[17] Wang C Zhai Z Yang Y Wu Q Cheng Z Liang L. Efficacy and safety of low dose recombinant tissue-type plasminogen activator for the treatment of acute pulmonary thromboembolism: a randomized, multicenter, controlled trial. Chest. (2010) 137:254–62. 10.1378/chest.09-0765 19741062 PMC7126994

[18] Kiser TH Burnham EL Clark B Ho PM Allen RR Moss M. Half-dose versus full-dose alteplase for treatment of pulmonary embolism. Crit Care Med. (2018) 46:1617–25. 10.1097/CCM.0000000000003288 29979222 PMC6375681

[19] Burns KEA McLaren A. Catheter-related right atrial thrombus and pulmonary embolism: a case report and systematic review of the literature. Can Respir J. (2009) 16:163–5. 10.1155/2009/751507 19851535 PMC2779172

[20] Alirezaei T Hajimoradi B Pishgahi M Nekooghadam SM Golmohamadi M. Successful systemic thrombolytic therapy for massive pulmonary embolism in a patient with breast cancer, brain metastasis, and thrombocytopenia: a case report. Clin Case Rep. (2018) 6:1431–5. 10.1002/ccr3.1629 30147877 PMC6099001

[21] Zhu T Pan K Wang Y. Successful resuscitation with thrombolysis of pulmonary embolism due to thrombotic thrombocytopenic purpura during cardiac arrest. Am J Emerg Med. (2015) 33:132.e3–e4. 10.1016/j.ajem.2014.06.025 25070193

[22] Hourmouzis Z Bhalla MC Frey JA Jwayyed S. Pulmonary embolism and heparin-induced thrombocytopenia successfully treated with tissue plasminogen activator and argatroban. Am J Emerg Med. (2015) 33:739.e5–e6. 10.1016/j.ajem.2014.11.027 25497698

[23] Iqbal P Habib MB Hatim A Alkhatib M Bakar MA Koya SH. Dilemma of thrombolysis in a patient with high-risk pulmonary embolism with severe thrombocytopenia: a case report. Qatar Med J. (2023) 2023:39. 10.5339/qmj.2023.39 38223832 PMC10787518

[24] Ahmadzadeh A Nazar Poor S Hamneshin Behbahani R Soltani P Tavasol A Sheibani M. Thrombolytic therapy for massive pulmonary embolism in an antiphospholipid syndrome patient with severe thrombocytopenia: a case report. Clin Case Rep. (2025) 13:e71395. 10.1002/ccr3.71395 41268293 PMC12626766

[25] Schulman S Kearon C. Subcommittee on control of anticoagulation of the SSC of the ISTH. Definition of major bleeding in clinical investigations of antihemostatic medicinal products in non-surgical patients. J Thromb Haemost. (2005) 3:692–4. 10.1111/j.1538-7836.2005.01204.x 15842354

[26] Mehran R Rao SV Bhatt DL Gibson CM Caixeta A Eikelboom J. Standardized bleeding definitions for cardiovascular clinical trials: a consensus report from the bleeding academic research consortium. Circulation. (2011) 123:2736–47. 10.1161/CIRCULATIONAHA.110.009449 21670242

[27] Valent P Klion AD Roufosse F Simon D Metzgeroth G Leiferman KM. Proposed refined diagnostic criteria and classification of eosinophil disorders and related syndromes. Allergy. (2023) 78:47–59. 10.1111/all.15544 36207764 PMC9797433

[28] Ray JG Kearon C Yi Q Sheridan P Lonn E. HOPE-2 investigators. Homocysteine-lowering therapy and risk for venous thromboembolism: a randomized trial. Ann Intern Med. (2007) 146:761–7. 10.7326/0003-4819-146-11-200706050-00157 17470822

